# Glyoxalase I activity affects *Arabidopsis* sensitivity to ammonium nutrition

**DOI:** 10.1007/s00299-022-02931-5

**Published:** 2022-10-15

**Authors:** Klaudia Borysiuk, Monika Ostaszewska-Bugajska, Katsiaryna Kryzheuskaya, Per Gardeström, Bożena Szal

**Affiliations:** 1 Department of Plant Bioenergetics, Institute of Experimental Plant Biology and Biotechnology, Faculty of Biology, University of Warsaw, Miecznikowa 1, 02-096 Warsaw, Poland; 2 Department of Plant Physiology, Umeå Plant Science Centre, Umeå University, 90187 Umeå, Sweden

**Keywords:** Ammonium nutrition, Dicarbonyl stress, d-Lactate dehydrogenase, Glyoxalase, Methylglyoxal, Mitochondrial Complex I mutant

## Abstract

**Key message:**

Elevated methylglyoxal levels contribute to ammonium-induced growth disorders in *Arabidopsis thaliana*. Methylglyoxal detoxification pathway limitation, mainly the glyoxalase I activity, leads to enhanced sensitivity of plants to ammonium nutrition.

**Abstract:**

Ammonium applied to plants as the exclusive source of nitrogen often triggers multiple phenotypic effects, with severe growth inhibition being the most prominent symptom. Glycolytic flux increase, leading to overproduction of its toxic by-product methylglyoxal (MG), is one of the major metabolic consequences of long-term ammonium nutrition. This study aimed to evaluate the influence of MG metabolism on ammonium-dependent growth restriction in *Arabidopsis thaliana* plants. As the level of MG in plant cells is maintained by the glyoxalase (GLX) system, we analyzed MG-related metabolism in plants with a dysfunctional glyoxalase pathway. We report that MG detoxification, based on glutathione-dependent glyoxalases, is crucial for plants exposed to ammonium nutrition, and its essential role in ammonium sensitivity relays on glyoxalase I (GLXI) activity. Our results indicated that the accumulation of MG-derived advanced glycation end products significantly contributes to the incidence of ammonium toxicity symptoms. Using *A. thaliana frostbite1* as a model plant that overcomes growth repression on ammonium, we have shown that its resistance to enhanced MG levels is based on increased GLXI activity and tolerance to elevated MG-derived advanced glycation end-product (MAGE) levels. Furthermore, our results show that glyoxalase pathway activity strongly affects cellular antioxidative systems. Under stress conditions, the disruption of the MG detoxification pathway limits the functioning of antioxidant defense. However, under optimal growth conditions, a defect in the MG detoxification route results in the activation of antioxidative systems.

**Supplementary Information:**

The online version contains supplementary material available at 10.1007/s00299-022-02931-5.

## Introduction

Exceptional plasticity characterizes plant metabolism, which is essential for their capacity to adapt to environmental constraints since plants are sessile. Despite the evolutionary pressure on the effectiveness and specificity of chemical reactions occurring inside the cell, in many processes toxic compounds are formed, the level of which must be strictly controlled. The effect of these compounds depends on the dose. At a low level, they can act as specific signaling molecule with physiological roles, whereas at high levels, they can lead to cellular damage. Reactive carbonyl species (RCS) and reactive oxygen species (ROS) are among those substances that have a dual destructive–constructive nature. The rate of their synthesis often increases due to metabolic pathway overloading or from an imbalance in cellular homeostasis, for example, too high cellular redox potential.

The RCS group includes many biological compounds with one or more carbonyl groups, which are constantly produced in all living organisms. RCS can be produced non-enzymatically in processes such as lipid peroxidation, and amino acid oxidation. They can also be derived enzymatically via the activity of enzymes involved in the glycolytic or the polyol pathway, among others (Niwa 1999; Pompliano et al. 1990). Dicarbonyl compounds, including glyoxal, methylglyoxal (MG), and 3-deoxyglucosone form a special group within the RCS. Their excessive production leads to a phenomenon called dicarbonyl stress, which is harmful to cells because of the formation of RCS adducts with biomolecules, such as proteins or nucleic acids (Rabbani and Thornalley 2015).

In vivo, MG is a dominant mediator of dicarbonyl stress (Rabbani et al. 2020a, b). Within cells, the greatest amount of MG is produced in the glycolytic pathway due to the spontaneous degradation of triose phosphates (TP), glyceraldehyde-3-phosphate (GAP) or dihydroxyacetone phosphate (DHAP) (Richard 1993; Rabbani et al. 2020a, b); or as a by-product of triosephosphate isomerase activity (TPI) (Richard 1991). Like other organisms, plants possess an efficient glutathione-dependent MG-detoxifying system of glyoxalases, consisting of lactoylglutathione lyase (glyoxalase I, GLXI) and S-2-hydroxyacylglutathione hydrolase (glyoxalase II, GLXII), to degrade MG to d-lactate (Thornalley 1990). d-lactate is further metabolized by d-lactate dehydrogenase (d-LDH) in the mitochondria to a non-toxic metabolite (pyruvate), which can supply electrons to the mitochondrial electron transport chain (mtETC) (Schertl and Braun 2014). Both glyoxalases are encoded by a small multigene family in *Arabidopsis*.

Although Mustafiz et al. (2011) showed that the *Arabidopsis* genome encodes 11 *GLXI*-like and five *GLXII*-like proteins, specific glyoxalase activity was observed for only three proteins from each group (GLXI.1-3 and GLXII.2, 4, 5, respectively) (Schmitz et al. 2017). d-LDH is encoded by a single gene in *Arabidopsis* (Engqvist et al. 2009). As an alternative to the classical glyoxalase pathway, glutathione-independent glyoxalase III (GLXIII), which converts MG directly to d-lactate, is also known (Kumar et al. 2021). *GLXIII* belongs to the DJ-1 protein superfamily, also known as *PARK7*, and its protein products are well characterized in mammalian tissues because they are linked to the development of Parkinson's disease (Lee et al. 2012; Kumar et al. 2021). In plants, the products of only some of the six *GLXIII/DJ-1* have been proven to have specific glyoxalase activity. However, this activity seems to be significantly lower than that of the classical glyoxalase pathway of the MG detoxification route (Kwon et al. 2013; Lewandowska et al. 2019).

The toxicity of MG toward proteins results from its physicochemical properties. MG, a carbonyl electrophile, is a strong amino acid (AA)-directed glycating agent, leading to the formation of a major plant derivative of arginine belonging to advanced glycation end products (AGE), MG-derived hydroimidazolone 1 (MG-H1) and at lower rate, argpyrimidine (Rabbani et al. 2020a, b). In addition, MG derivatives of lysine, such as *N*^ε^-(1-carboxyethyl)lysine (CEL) or *N*,*N*(-di(*N*^ϵ^-lysino))-4-methyl-imidazolium (methylglyoxal-lysine dimer, MOLD) are also produced in plant tissues (Rabbani et al. 2020a, b). Since MG targets arginine and lysine,-AAs, which have profound roles in maintaining proper protein structure and function (Armstrong et al. 2016), the introduction of cross-links to protein molecules may result in the aggregation of misfolded proteins (Rabbani et al. 2020a, b). The level of AGE, including MG-derived AGE (MAGE) in cells or tissues, depends not only on MG production and the efficiency of its detoxification but also on the enzymatic activity of deglycases repairing MG-glycated amino acids and proteins (Richarme et al. 2015). In addition to AA damage, MG also has detrimental effects on nucleic acids (Thornalley et al. 2010), principally leading to the formation of imidazopurinone derivatives (Waris et al. 2015).

Oxidative damage of biomolecules, including amino acid residues in cellular protein structures, is also caused by ROS, which possess relatively high reactivity. ROS are produced in plant tissues as the products of specific enzymes or as unintended metabolites produced by the operation of electron transport chains, such as mitochondrial and chloroplastic transport chains (mtETC and chlETC, respectively). The synthesis of ROS increases especially when cells are exposed to excess reductants.

Under non-stress conditions, cellular ROS levels are maintained by enzymatic and non-enzymatic antioxidative systems. The former system includes superoxide dismutases (SOD), ascorbate peroxidases (APX), and other enzymes of the Foyer**–**Halliwell–Asada cycle or glutathione peroxidase-like proteins (GPX-like), whereas the latter system includes ascorbate, glutathione, tocopherol, cysteine, and others. Particularly prone to modification by ROS are sulfur-containing amino acids. Most of the ROS-dependent modifications at early stages of the oxidation process are reversible (Rey and Tarrago 2018) and vary in plant tissues depending on growth conditions (Bechtold et al. 2009). Therefore, such protein modification might have physiological relevance. In contrast, the carbonylation of proteins is an irreversible process and is considered a marker of oxidative damage (Møller et al. 2011). Carbonyl derivatives are mainly formed by a direct metal-catalyzed oxidative attack (MCO) on the amino acid side chains of proline, arginine, lysine, and less frequently, threonine (Nyström 2005); this process is called primary protein carbonylation. In addition, proteins can undergo secondary carbonylation, which refers to the modification of amino acid side chains (mainly on lysine, cysteine, and histidine residues) by adduct formation with lipid peroxidation-derived RCS formed by the oxidation of polyunsaturated fatty acids (PUFA) by ROS (Tola et al. 2021).

An increasing number of studies have confirmed that MG and ROS metabolism are functionally linked. A direct and dose-dependent effect of higher ROS production upon methylglyoxal treatment was first shown in rat hepatocytes (Kalapos et al. 1993). Subsequently, specific reactions of MG production and catabolism, resulting in increased ROS production, have been described (Kalapos 2008). Glutathione (GSH) is the common element in both ROS- and MG-detoxifying systems. Therefore, increased GSH involvement in MG detoxification may impair GSH-dependent ROS detoxification. The relationship between the metabolism of MG and ROS may also be linked indirectly through ROS-responsive regulation of enzyme activity leading to MG production (de Bari et al. 2020 and references therein). Using an animal experimental system, it was proven also that proteins classified as components of the antioxidant system and pro-oxidant enzymes might be inactivated or activated, respectively, by MG-dependent modifications (Kang 2003; Chang et al. 2005). The inactivation of specific antioxidant enzymes by MG has also been observed in plants (Hoque et al. 2010, 2012).

Irreversible modifications of amino acid side chains by MG and ROS have a devastating effect on the structure and activity of proteins and thus on cell functioning. The accumulation of injured proteins triggers their degradation (Nyström 2005) by the ubiquitin–proteasome system (Kurepa et al. 2008) and when cytotoxic aggregates are formed, degradation occurs by aggrephagy (Jung et al. 2020).

Most abiotic stresses lead to increased ROS production (Sharma et al. 2012; Das and Roychoudhury 2014), but only some of them have been tested to evaluate if their action leads to increased production of MG (Nahar et al. 2015; Melvin et al. 2017; Gupta et al. 2018). Previously, we have shown that ammonium stress leads to an imbalance in the oxidation–reduction status of leaf cells, resulting in increased ROS production (Podgórska et al. 2013, 2018a, b). Moreover, we have reported that in *Arabidopsis* ammonium nutrition leads to enhancing of the glycolytic pathway that promotes MG production (Borysiuk et al. 2018). Both ROS- (Podgórska et al. 2015) and MG-dependent (Borysiuk et al. 2018) damage to biomolecules may contribute to ammonium syndrome symptoms.

However, other metabolic cues, such as energy deficiency (Podgórska et al. 2013), shoot carbohydrate limitation (Schortemeyer et al. 1997) or ion (Van Beusichem et al. 1988), and phytohormone (Walch-Liu et al. 2000; Dziewit et al. 2021) imbalance are also important in promoting ammonium-induced developmental disorders. To determine the extent to which MG metabolism influences ammonium-dependent growth restrictions in *Arabidopsis* plants, we analyzed the growth and parameters of MG-related metabolism in plants with dysfunction in the glyoxalase pathway grown under conditions of ammonium nutrition. We were also interested in determining the relationship between ROS and MG metabolism upon ammonium treatment. Previously, it was shown that the *Arabidopsis* mutant *frostbite 1* (*fro1*), characterized by the lack of mitochondrial Complex I activity, had improved resistance to ammonium stress (Podgórska et al. 2015). Based on this finding, we were also interested in verifying whether differences in ammonium sensitivity of *fro1* plants are due to alterations in MG metabolism. We have shown that MAGE accumulation is a significant reason for ammonium toxicity symptoms, and enhancement of ammonium resistance of *fro1* plants may rely on tolerance to their elevated level. Besides, we have documented that GLXI.3 capacity plays a crucial role in ammonium stress resistance. Our results also indicate that the functioning of the enzymatic antioxidant system is significantly disrupted in *glxI.3* mutants grown in the presence of ammonium ions.

## Materials and methods

### Plant material

*Arabidopsis thaliana*, wild-type ecotype Columbia-0 (Col-0), and C24 were used as the plant materials in the experiments. In the Col-0 background, the insertional lines with the disruption of specific genes involved in MG detoxification used in the experiments were purchased from Nottingham Arabidopsis Stock Center (NASC), including SALK_035429 (*glxI.3_429*, NASC ID N535429), SALK_131547 (*glxI.3_547*, NASC ID N631547), SALK_014288 (*glxII.5_288*, NASC ID N514288), SALK_073585C (*glxII.5_971*, NASC ID N662971), SALK_054500 (*dldh*_*500*, NASC ID 554500), SALK_026859C (*dldh_520*, N685520), SALK_000803 (*dj-1a*, NASC ID N500803), SALK_093414 (*dj-1b*, NASC ID N593414), and SALK_111176 (*dj-1d*, NASC ID N611176)*.* Homozygous lines of the insertional mutants were derived after PCR-based genotyping using gene-specific primers (Supplementary Table 1 and Supplementary Figure 1). Analyses were also performed using a *fro1* mutant that was derived through chemical mutagenesis of the C24 ecotype of *Arabidopsis* (Lee et al. 2002). *Fro1* plants possess a single point mutation in the nuclear-encoded 18-kDa Fe–S subunit (NDUFS4) of mitochondrial Complex I, which confers a G-to-A change at an intron–exon junction at the start codon resulting in mis-splicing and a premature stop codon (Lee et al. 2002). Plants with truncated NDUFS4 protein are unable to assemble Complex I and are characterized by a dwarf phenotype, disrupted redox and energy metabolism but enhanced resistance to ammonium (Podgórska et al. 2015).

### Long-term plant growth conditions

For long-term ammonium treatment, plants were grown using the Araponics SA growth systems (Araponics, Liège, Belgium) designed for *Arabidopsis* hydroponic cultures. Briefly, seedlings, after germination in holders filled with ½ Murashige and Skoog (1962) (MS, M5524, Merck, Darmstadt, Germany) solidified with 1% agar, were grown on a liquid medium composed of 1.5 mM KH_2_PO_4_, 2.5 mM KCl, 0.7 mM CaSO_4_·2H_2_O, 0.8 mM MgSO_4_·7H_2_O, 0.06 mM NaFe-EDTA, 5 mM CaCO_3_, and micronutrient solution (0.20 μM CuSO_4_·5H_2_O, 0.35 μM ZnSO_4_·7H_2_O, 8.90 μM H_3_BO_3_, 0.15 μM Al_2_(SO_4_)_3_, 2 nM MnCl_2_·4H_2_O, 0.20 μM NiSO_4_·6H_2_O, 0.17 μM Co(NO_3_)_2_·6H_2_O, 0.15 μM KI, 0.21 μM KBr, 0.21 μM Na_2_MoO_4_·2H_2_O), and 2.5 mM Ca(NO_3_)_2_·4H_2_O or 2.5 mM (NH_4_)_2_SO_4_ as the nitrogen source as described in Podgórska et al. (2015). The growth medium was aerated and replaced twice a week. Plants were stored in growth chambers under short day-light conditions (8 h/16 h), 150 μmol m^−2^ s^−1^ photosynthetically active radiation (PAR), and day/night temperatures of 21 °C/18 °C. *Arabidopsis* was grown under the same conditions as in the paper by Borysiuk et al. (2018) in which up-regulation of methylglyoxal metabolism under conditions of ammonium nutrition was previously proven. Randomly selected rosettes from two independent nitrate or ammonium plant cultures were weighed to determine their fresh weight (FW). The leaves were cut and immediately frozen in liquid nitrogen and then kept at −80 °C to later determine metabolite levels or enzyme activities.

### In vitro seedling cultivation and treatment with glyoxalase inhibitor, d-lactate, or MG

For in vitro assays, *Arabidopsis* seeds were surface sterilized with 70% ethanol for 5 min and then with 1% sodium hypochlorite solution for 5 min and subsequently rinsed three times with sterile deionized water. The seeds were then sown on agar-solidified growth media and kept in the dark for 48 h at 4 °C for seed stratification. In vitro* Arabidopsis* growth was performed in growth chambers under long day-light conditions (16 h/8 h), and other growth parameters were the same as those for long-term treatment. Root growth tests were performed using a vertical-plate agar assay. Seeds were sown on modified Somerville medium composed of 2.5 mM KH_2_PO_4_, 2 mM MgSO_4_, 50 µM ferric-ethylenediaminetetraacetic acid (Fe-EDTA), and 2.5 mM Ca(NO_3_)_2_ or 2.5 mM (NH_4_)_2_SO_4_ as a nitrogen source. In the ammonium-containing media, calcium ions were added as 2.5 mM CaCl_2_. Media were supplemented with 5 mM 3-(*N*-morpholino)propane sulfonic acid (MOPS), pH 5.8, 1% (w/v) sucrose (Suc), and 0.8% agar, and in specific experiments with d-lactate or MG at concentrations of 5 mM or 2.5 mM, respectively.

Root length was measured after 7 days of seedling growth under light conditions. Sterile seeds were embedded in 0.1% agar solution and germinated on agar-solidified ½ MS medium supplemented with 1% Suc to test the sensitivity to GLXI inhibitor (Thornalley et al. 1996). Seven-day-old seedlings were transferred into multi-well plates filled with liquid Somerville medium deprived of nitrogen. After a further 14 h, the wells were filled with full Somerville medium containing nitrate or ammonium as the nitrogen source supplemented with S-*p*-bromobenzylglutathione cyclopentyl diester (BBGD, SML1306, Merck) (Thornalley et al. 1996), while the control wells did not include inhibitor. Phenotypic disorders were imaged in a shadeless tent (Puluz, Shenzen, China) after 72 h of treatment with GLXI inhibitor. For MG treatment, seedlings were grown on MS medium supplemented with 1% Suc until the 1.02–1.04 growth stage, according to Boyes et al. (2001), and then transferred to a liquid medium consisting of MS salts, 5 mM MOPS (pH 5.8), and 1% Suc supplemented with MG. The effect of MG on leaf seedling phenotype was imaged after 24 h of treatment.

### Enzyme activity assays

GLXI was extracted from leaf tissues, according to Chakravarty and Sopory (1998). GLXI activity was measured as the formation of *S*-d-lactoylglutathione (SLG) from hemithioacetal, as described by Dafre et al. (2015) with modifications. Hemithioacetal was freshly obtained by the non-enzymatic reaction of 2 mM MG with 2 mM GSH in a 50 mM potassium phosphate-buffered solution. The GLXI reaction was stopped after 20 min by adding perchloric acid (final concentration, 1 M). The absorbance of the samples was read at *λ* = 240 nm, and activity was calculated using the absorbance coefficient for SLG 3.37 mM^−1^ cm^−1^. All measured values were corrected using values obtained in parallel control experiments (assays with samples treated by PCA prior to adding the reaction substrate). GLXII activity assay extracts were prepared as described by Singla-Pareek et al. (2006). GLXII activity was determined by the reaction of 5,5′-dithio-bis-2-nitrobenzoic acid (DTNB) with GSH released from SLG (Martins et al. 1999). d-LDH was measured in the cytochrome *c* reduction reaction, as described by Schertl et al. (2014). Protease activity was assayed by the degradation of resorufin-labeled casein, as described by Borysiuk et al. (2018). TPI activity was measured using the method described by Ito et al. (2003) and Ostaszewska-Bugajska et al. (2015). The non-phosphorylating NADP^+^-dependent glyceraldehyde-3-phosphate dehydrogenase (NADP^+^-GAPDH) activity was assayed as described by Bustos and Iglesias (2003).

### Measurements of metabolite levels

MG levels in the leaf tissue were estimated as described by MacWilliams et al. (2020). The protein content in the samples was determined using the *RC DC*™ Protein Assay kit (Bio-Rad Laboratories, Hercules, CA, USA) or Bradford reagent (B6916, Merck).

### Tissue antioxidant capacity determination

Leaf samples (50 mg) were homogenized with 150 µL of cooled phosphate-buffered saline (1 × PBS) using a bead mixer mill (MM 400, Retsch, Haan, Germany). The supernatant collected after centrifugation (10 min, 15,000*g*) diluted tenfold was used in the assay. Low-weight antioxidant capacity and protein antioxidant capacity was determined using Total Antioxidant Capacity Assay Kit (MAK187, Merck) according to the manufacturer’s recommendations.

### Mitochondria isolation and mitochondrial oxygen uptake

Crude leaf mitochondria were isolated as described by Keech et al. (2005) and purified using a discontinuous Percoll gradient density as previously described by Podgórska et al. (2015). The oxygen consumption rate of purified mitochondria was measured polarographically using a Clark-type electrode system (Oxygraph and Oxygraph Plus Software; Hansatech, Norfolk, England) in an incubation medium containing 0.45 M mannitol, 10 mM phosphate buffer (pH 7.2), 5 mM MgCl_2_, 10 mM KCl, and 0.1% (w/v) bovine serum albumin (BSA) in the presence of 5 mM d-lactate as the substrate as was described in Borysiuk et al. (2018).

### Western blot analyses

The level of MG-H1 was determined after standard procedures of sodium dodecyl sulfate–polyacrylamide gel electrophoresis (SDS–PAGE) using a 14% resolving gel and wet transfer onto polyvinylidene difluoride (PVDF) membranes of leaf samples (15 µg protein loaded per lane). Membranes were immunolabeled with primary anti-MG-H1 (STA 011, Cell Biolabs, San Diego, CA, USA). For the assay of carbonylated protein levels, leaf protein samples were derivatized with 2,4-dinitrophenylhydrazine (DNPH), as described by Ostaszewska-Bugajska et al. (2022), and then loaded on 10% polyacrylamide gels (5 µg per lane). The standard protocol for SDS-PAGE and semi-dry electroblotting was then applied. Immunolabeling was performed using polyclonal primary anti-DNP antibodies at a dilution of 1:1000 (D9656, Merck). The leaf extracts were subjected to SDS-PAGE (20 µg of protein per lane), electroblotted onto a nitrocellulose membrane, and probed overnight at 4 °C with primary antibodies anti-APX (AS08368 Agrisera, Vännäs, Sweden) and anti-GR (AS06181, Agrisera) diluted according to the manufacturers’ instructions, to determine APX and GR levels. Membranes tagged with primary antibodies were incubated with anti-mouse (for MG-H1) or anti-rabbit (for carbonylated proteins and ROS-related proteins) secondary antibodies conjugated to horseradish peroxidase. Immunolabeled proteins were visualized using a chemiluminescence kit (Clarity Western ECL; Bio-Rad). Signals were detected using a ChemiDoc imaging system (Bio-Rad Laboratories). The staining intensity of bands (GR, APX) or the entire blot lane (MG-H1, carbonylated proteins) was quantified by densitometry using Image-Lab 5.2 software (Bio-Rad Laboratories) after background correction.

### Quantitative reverse transcription-polymerase chain reaction analysis

RNA isolation, cDNA synthesis, and qPCR assays were performed as described by Borysiuk et al. (2018). Transcript abundance was normalized to the transcript level of the reference gene *PROTEIN PHOSPHATASE 2A* (*PP2A*, AT1G13320). Quantification of mRNA and qRT-PCR efficiency of the target genes was performed as described by Pfaffl (2001). Transcript levels were expressed relative to the genotype with the lowest expression in nitrate-grown plants (value of 1). The primer sequences to measure the transcript abundance of *GLXI.3* (AT1G08110), *GLXII.2* (AT3G10850), *GLXII.4* (AT1G06130), and *GLXII.5* (AT2G31350) were described by Borysiuk et al. (2018). New primers designed for *D-LDH* (AT5G06580), *DJ-1A* (AT3G14990), *DJ-1B* (AT1G53280), *DJ-1D* (AT3G02720), *DJ-1E* (AT2G38860), and *DJ-1F* (AT3G54600) used in experiments are listed in Supplementary Table 2.

### Statistical analysis

Values are the mean ± standard deviation (SD) of three to eleven independent biological replicates (*n*). Protein gel blot analysis was performed using data from at least two independent experiments, with a minimum of three biological replicates. To analyze the statistical significance of the observed differences, one-way analysis of variance (ANOVA) with Tukey’s post hoc test was performed using Statistica 13.3 software (StatSoft, Inc., Tulsa, OK, USA). Bars or means denoted by different letters indicate significant differences between treatments and genotypes (*p* ≤ 0.05).

## Results

### The impact of disruption of glyoxalase pathway on plant sensitivity to ammonium nutrition

**Fig. 1:**
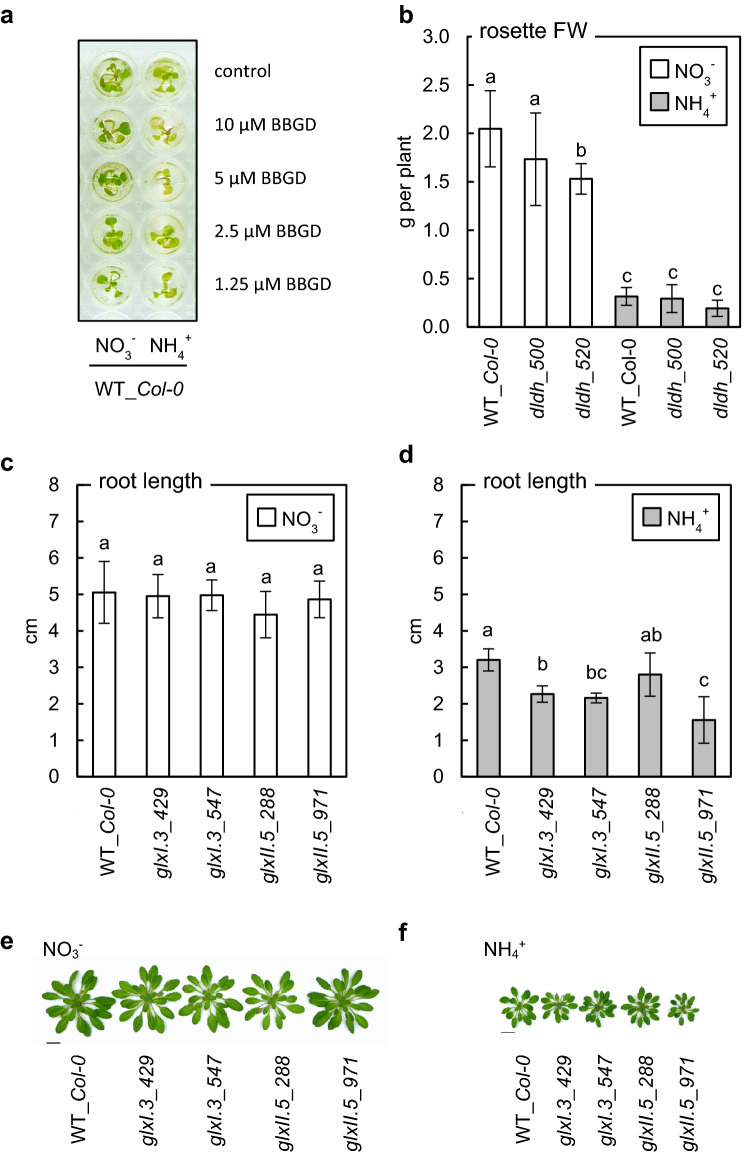
Influence of impairment of methylglyoxal and d-lactate detoxification pathways on the phenotype of *Arabidopsis* grown on nitrate (NO_3_^−^) or ammonium (NH_4_^+^) as the sole nitrogen source. Treatment of wild-type (WT_*Col-0*) seedlings with S-*p*-bromobenzylglutathione cyclopentyl diester (BBGD) (**a**), fresh weight (FW) of rosettes of long-term grown d-lactate dehydrogenase insertion lines (*dldh*) (mean ± SD; *n* = 8–9) (**b**) and the length of seedlings roots of glyoxalase I.3 (*glxI.3*) and II.5 (*glxII.5*) insertion lines in a vertical-plate agar assay using nitrate- (**c**) or ammonium-contained (**d**) medium (mean ± SD; *n* = 10–11). Phenotype of long-term grown glyoxalase I.3 (*glxI.3*) and glyoxalase II.5 (*glxII.5*) insertion lines cultivated on nitrate- (**e**) or ammonium-supplied (**f**) medium. Scale bar represents 2 cm. Representative photos are shown. Statistically significant differences by ANOVA (*p* ≤ 0.05) with Tukey’s post hoc test are indicated by different letters above the bars

Our previously published results suggested that MG-related injury might be a key factor leading to ammonium syndrome (Borysiuk et al. 2018). Therefore, the efficiency of the glyoxalase pathway may be one of the determining factors of plant sensitivity to ammonium. To test this hypothesis, we examined the response of *Arabidopsis* seedlings grown under different nitrogen regimes to the GLXI inhibitor, BBGD. Application of BBGD led to more pronounced phenotypic disorders in ammonium-grown plants than in nitrate-grown plants (Fig. 1a). This observation confirmed that the glyoxalase detoxification pathway was crucial for plant development under ammonium nutrition conditions. However, to the best of our knowledge, BBGD has not been tested in plant systems and may have a non-specific effect on plant metabolism. Therefore, to test our hypothesis, insertional mutants with disruption of the glyoxalase pathway were used. Since the enhancement of the glyoxalase pathway under ammonium nutrition was previously linked mainly to increased GLXI.3 or GLXII.5 transcript level (Borysiuk et al. 2018) related to cytosolic/chloroplastic and mitochondrial/chloroplastic isoform of GLX, respectively, the transformants *glxI.3* and *glxII.5* were used in further experiments.

Under long-term growth conditions the biomass of rosettes with impaired *D-LDH* expression was similar to that of the control plants at both nutrition regimes (Fig. 1b). Further, glyoxalase pathway dysfunction, either due to *GLXI.3* or *GLXII.5* disruption, had no effect on the roots length of seedlings grown on nitrate-containing medium (Fig. 1c). However, under ammonium treatment, the impairment of *GLXI.3* resulted in a decrease in the length of roots by approximately 30–35% (Fig. 1d). Measurements of the root lengths of *glxII.5* lines did not give clear results about the role of this protein under ammonium feeding conditions. The root lengths of the *glxII.5_288* line were similar to those of the control line, whereas those of *glxII.5_971* were lowered by approximately 50% (Fig. 1d). Similar trends were observed for shoot of long-term grown mature plants. Nitrate nutrition did not cause the differences in rosette size of the tested insertion lines as compared to the control (Fig. 1e), while under ammonium nutrition, the rosette size of *glxI.3_429*, *glxI.3_547*, and *glxII.5_971* was reduced.

**Fig. 2:**
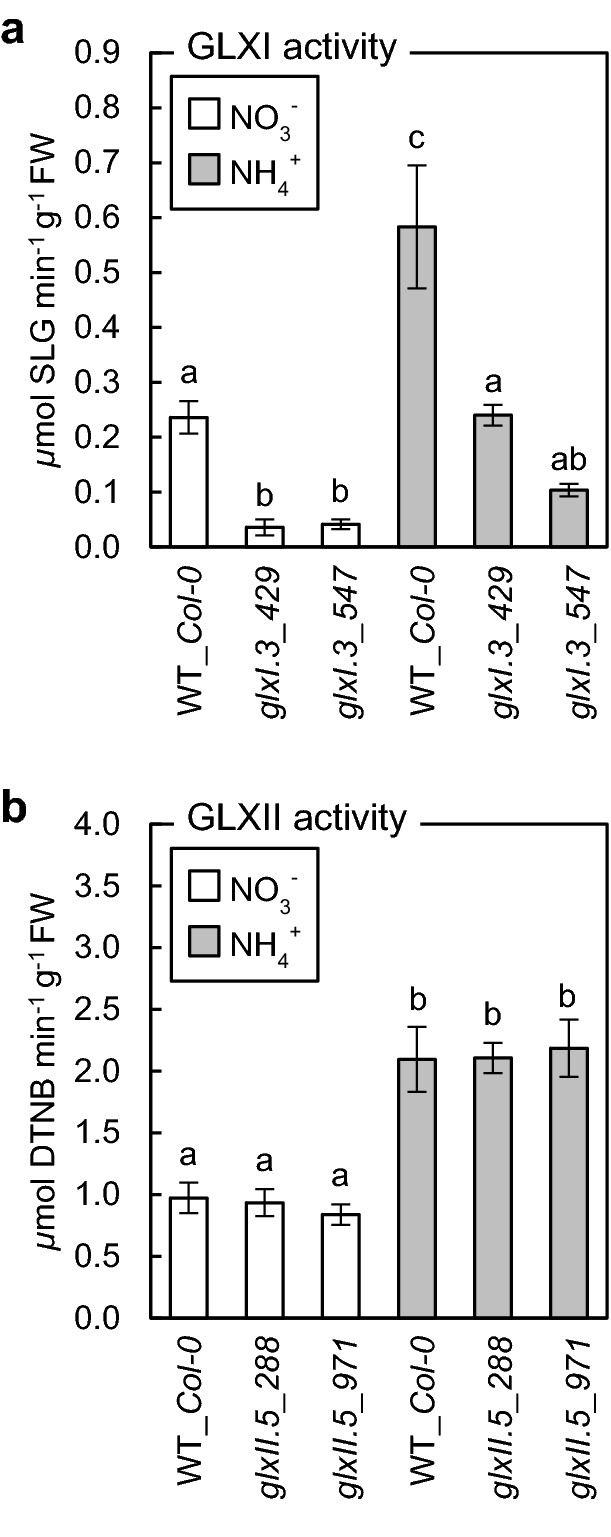
Impact of glyoxalase gene disruption on the corresponding glyoxalase activity in *Arabidopsis* plants long-term grown on nitrate (NO_3_^−^) or ammonium (NH_4_^+^) as the sole nitrogen source. Glyoxalase I (GLXI) activity in glyoxalase I.3 (*glxI.3*) insertion lines (**a**) and glyoxalase II (GLXII) activity in glyoxalase II.5 (*glxII.5*) insertion lines (**b**) as compared to wild-type (WT_*Col-0*) plants. Data are presented as mean ± SD (*n* = 3). Statistically significant differences by ANOVA (*p* ≤ 0.05) with Tukey’s post hoc test are indicated by different letters above the bars

Because both GLXI and GLXII are encoded by multigene families, we analyzed whether disruption of the expression of individual genes corresponds to lowered total enzyme activity. Indeed, *GLXI.3* disruption resulted in a massive decrease in GLXI activity, and GLXI activity in both *glxI* transformants was inhibited by approximately 70% under nitrate and by 60–80% under ammonium nutrition (Fig. 2a). In contrast, disruption of *GLXII.5* did not influence the total GLXII activity in mutant lines compared to the control lines under either nitrate or ammonium growth conditions (Fig. 2b).

Overall, the activities of both glyoxalases were elevated or tended to be higher in response to ammonium stress in all the tested genotypes (Fig. 2a, b). Taken together, these results confirm that glyoxalase pathway activity is important in determining plant sensitivity toward ammonium nutrition. Moreover, since GLXI activity in *glxI.3* was significantly decreased (Fig. 2a), our results indicated that disruption of *GLXI.3* expression cannot be compensated by enhanced expression of other GLXI isoforms under ammonium stress. In contrast, we noticed that even though *GLXII.5* is upregulated by ammonium (Borysiuk et al. 2018) when the lack of this isoform is genetically enforced, compensation with other GLXII isoforms occurs, and no differences in GLXII activity were observed in *glxII.5* plants (Fig. 2b).

### Role of DJ-1 proteins under ammonium stress

**Fig. 3:**
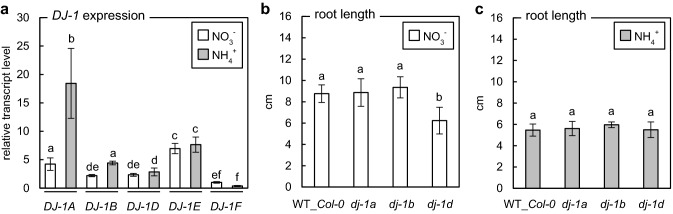
Role of DJ-1 proteins in *Arabidopsis* grown on nitrate (NO_3_^−^) or ammonium (NH_4_^+^) as the sole nitrogen source. Relative transcript level of DJ-1 genes in 9-week-old wild-type (WT_*Col-0*) plants (mean ± SD; n = 3) (**a**). Root length of 7-day-old *dj-1* seedlings grown on nitrate- (**b**) or ammonium-contained (**c**) medium in a Petri dish experiment (mean ± SD; *n* = 9–10). Statistically significant differences by ANOVA (*p* ≤ 0.05) with Tukey’s post hoc test are indicated by different letters above the bars

Since classical glyoxalase pathway activity might be supported by the glyoxalase activity of DJ-1 proteins, we evaluated the changes in the expression of genes belonging to the *Arabidopsis* GLXIII family in response to different nitrogen statuses. The expression of *DJ-1A* and *DJ-1B* genes was upregulated in response to ammonium, whereas *DJ*-*1D*, *DJ*-*1E*, and *DJ*-*1F* expression was not ammonium responsive (Fig. 3a). Furthermore, we determined the influence of disruption of *GLXIII* expression on plant growth. We did not observe any changes in the length of roots of the *dj-1* transformants, except for *dj-1d* plants, which were significantly shorter when nitrate was a component of the growth medium (Fig. 3b, c). These results suggest that DJ-1 protein activity is not a significant factor in modifying plant sensitivity to ammonium nutrition.

### MG-related protein damage in plants with glyoxalase pathway dysfunction under ammonium nutrition

**Fig. 4:**
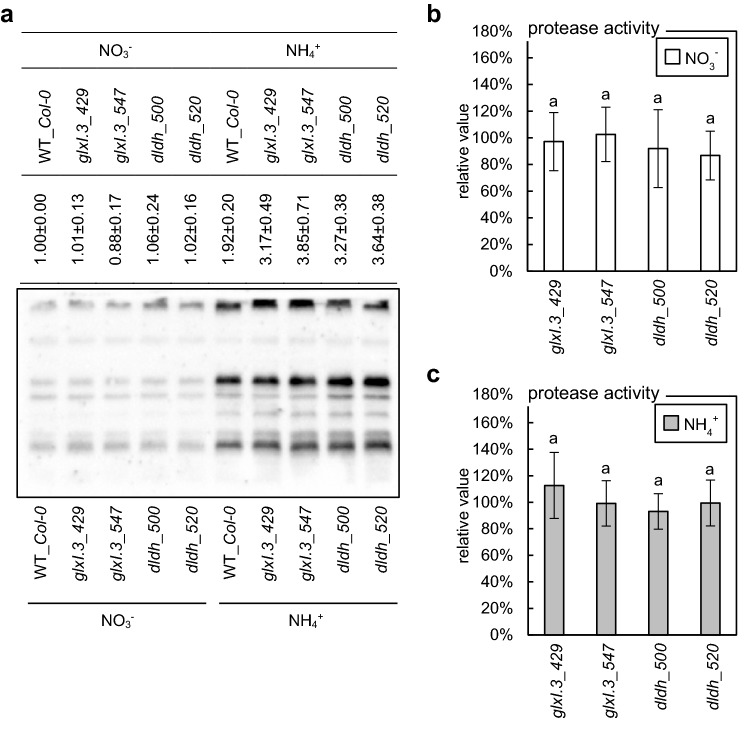
Influence of disabled methylglyoxal and d-lactate detoxification pathways on advanced glycation end-product formation and protease activity in *Arabidopsis* plants long-term grown on nitrate (NO_3_^−^) or ammonium (NH_4_^+^) as the sole nitrogen source. The level of methylglyoxal-derived hydroimidazolone 1 (MG-H1) (**a**) and the activity of proteolytic enzymes in d-lactate dehydrogenase (*dldh*) and glyoxalase I.3 (*glxI.3*) insertion lines grown under nitrate (**b**) or ammonium (**c**) conditions (mean ± SD; *n* = 3–4). The signal intensity for the whole lanes quantified by densitometry is given in the table above the representative protein gel blot (mean ± SD; *n* = 4). The fold changes were calculated relative to the value for wild-type (WT_*Col-0*) plants, which is presented as 100% (protease activity) or 1 (blot). Statistically significant differences by ANOVA (*p* ≤ 0.05) with Tukey’s post hoc test are indicated by different letters above the bars

To confirm that ammonium nutritional sensitivity may be related to the increased level of MG-related protein damage, we estimated the level of MG-H1 modification. No differences between WT and plants with dysfunction of glyoxalases or d-LDH were observed under nitrate conditions, whereas ammonium nutrition led to the accumulation of the main MAGE form in plants with disruption of the MG detoxification route (Fig. 4a). Since proteases catalyze the degradation of damaged proteins, their activity may be perceived as a protective mechanism when MG-dependent injuries occur. Further, the level of defective proteins is a balance between processes of damage and elimination. Therefore, we included measurements of protease activity in our experiments. However, an increased level of irreversibly modified proteins arising from MG toxicity in insertional mutants (Fig. 4 a) did not modify the protease activity (Fig. 4b, c).

### ROS metabolism in plants with enhanced MG production

**Fig. 5:**
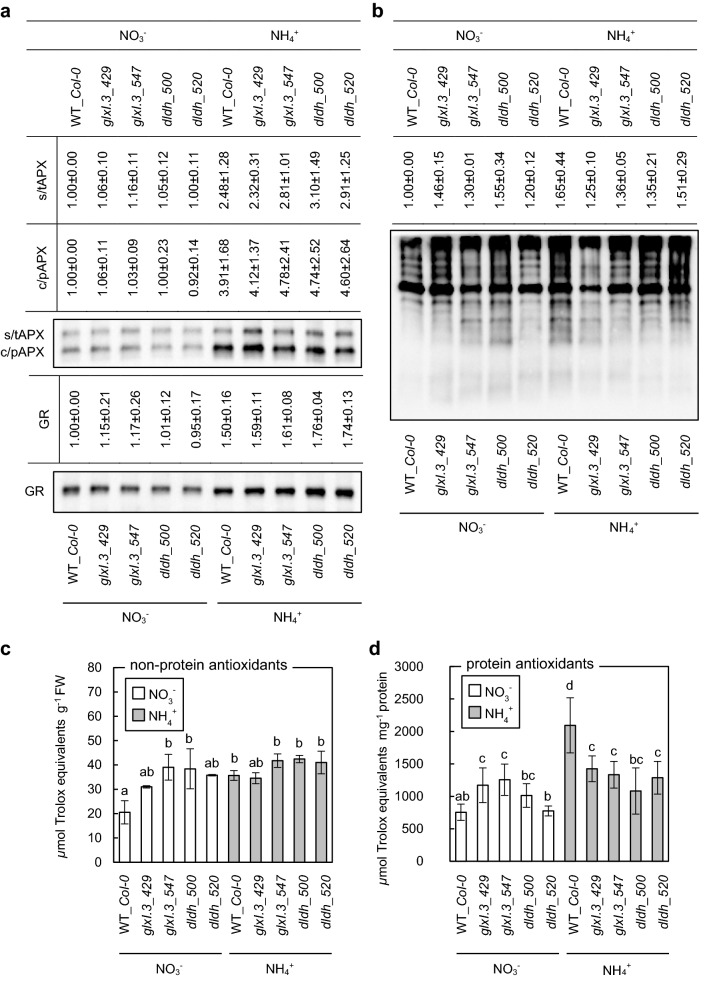
Cellular antioxidative defense and oxidative damage to proteins in *Arabidopsis* plants grown on nitrate (NO_3_^−^) or ammonium (NH_4_^+^) as the sole nitrogen source. The protein level of ascorbate peroxidase (APX) isoforms and glutathione reductase (GR) (**a**), the profile of protein carbonylation in leaf tissues (**b**). The capacity of non-protein antioxidant systems (**c**) and enzymatic antioxidant systems (**d**) in d-lactate dehydrogenase (*dldh*) and glyoxalase I.3 (*glxI.3*) insertion lines (mean ± SD; *n* = 3). Two chloroplastic APX isoforms, stromal and thylakoidal APX (s/tAPX) or cytosolic and peroxisomal (c/pAPX), are shown. The signal intensity for band (GR and APX) (mean ± SD; *n* = 4) or the whole lane (carbonylated proteins) (mean ± SD; *n* = 3) quantified by densitometry is given in the table above the representative protein gel blot. The fold changes on blots were calculated relative to the value for wild-type (WT_*Col-0*) plants grown on NO_3_^−^, which is presented as 1. Statistically significant differences by ANOVA (*p* ≤ 0.05) with Tukey’s post hoc test are indicated by different letters above the bars

In our previous research, we proved that long-term ammonium nutrition is linked to oxidative stress (Podgórska et al. 2013, 2015). It was also shown that impairment of antioxidant enzymes by MG treatment occurs (Hoque et al. 2010, 2012). We tested whether the increased ammonium sensitivity of plants with enhanced MG production was mainly due to MAGE formation or general MG-related dysfunction of antioxidant systems. In our experiments, we did not observe any influence of the impairment of the MG detoxification route on APX and GR protein levels (Fig. 5a). However, determination of the total antioxidant capacity of leaf tissues (Fig. 5c, d) revealed that enzymatic and non-protein antioxidant systems of the *glx1.3* and *dldh* mutants grown on nitrate were enhanced, apart from the enzymatic system in the *dldh_520* line. In contrast, a trend of reduced capacity of the enzymatic antioxidant system in leaf tissues of ammonium-grown insertional mutants was observed when compared to WT plants (Fig. 5d). The non-protein antioxidant system capacity under ammonium nutrition in insertional lines was unchanged when compared to WT plants (Fig. 5c). Mutations in the MG detoxification pathway did not affect the levels of carbonylated proteins when compared to WT plants (Fig. 5b).

### MG metabolism in *fro1* plants

**Fig. 6:**
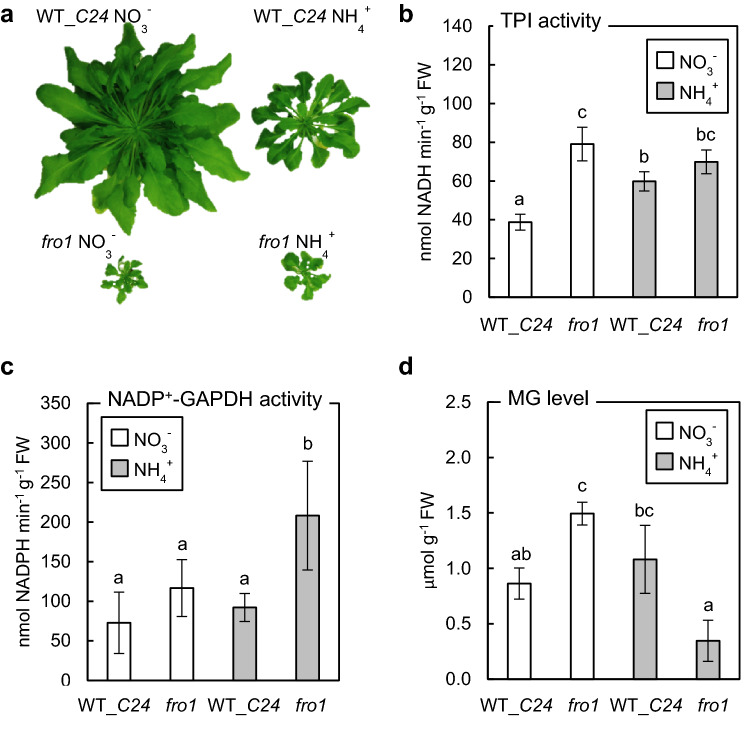
Methylglyoxal production in *Arabidopsis* plants overcoming ammonium syndrome that were long-term grown on nitrate (NO_3_^−^) or ammonium (NH_4_^+^) as the sole nitrogen source. Phenotype (**a**), triosephosphate isomerase (TPI) (mean ± SD; *n* = 4) (**b**), and non-phosphorylating NADP^+^-dependent glyceraldehyde 3-phosphate dehydrogenase (NADP^+^-GAPDH) (mean ± SD; *n* = 3–4) (**c**) activities as well as methylglyoxal (MG) level (mean ± SD; *n* = 3–5) (**d**) in *frostbite1* (*fro1*) as compared to wild-type (WT_*C24*)). Statistically significant differences by ANOVA (*p* ≤ 0.05) with Tukey’s post hoc test are indicated by different letters above the bars

Since *fro1* plants do not exhibit typical ammonium-induced growth inhibition (Fig. 6a), which is a major manifestation of ammonium syndrome, we analyzed whether the Complex I dysfunction resulted in modification of MG metabolism under ammonium nutrition compared to WT plants. Dysfunction of mtETC is linked with the upregulation of glycolytic flux (Kühn et al. 2015; Maclean et al. 2018); therefore in *fro1* plants, a > twofold increase in TPI activity, catalyzing the interconversion between DHAP and GAP, was observed when cultured on nitrate-containing medium as compared to WT plants. However, growth on ammonium led to enhanced TPI activity in WT plants but not in *fro1* plants (Fig. 6b). The activity of non-phosphorylating NADP^+^-GAPDH, constituting an alternative plant glycolytic route for the subsequent oxidation of the arising GAP, was highly induced only in *fro1* under ammonium nutrition in comparison to WT plants (Fig. 6c).

**Fig. 7:**
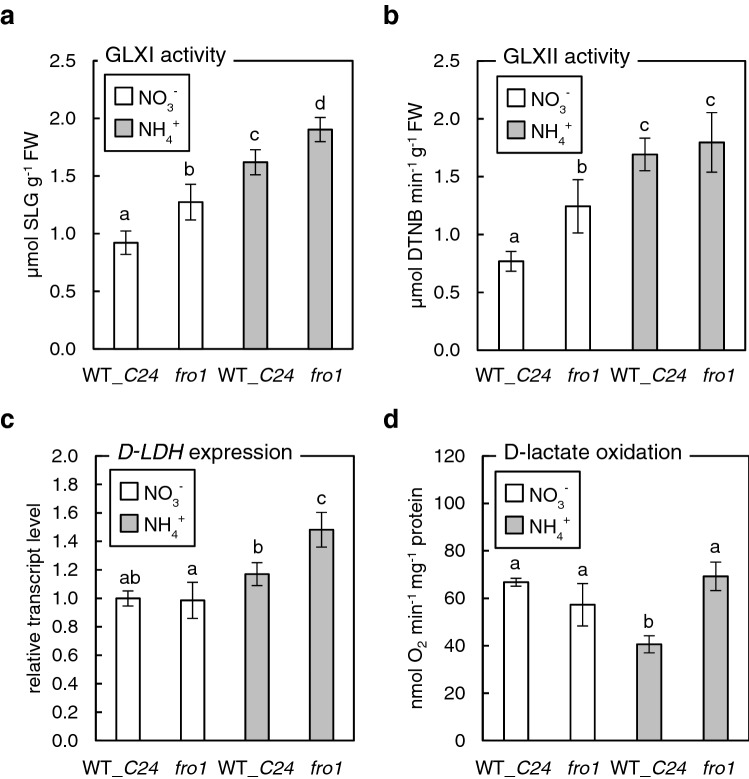
Methylglyoxal and d-lactate detoxification routes in *Arabidopsis* plants overcoming ammonium syndrome that were grown long-term on nitrate (NO_3_^−^) or ammonium (NH_4_^+^) as the sole nitrogen source. Glyoxalase I (GLXI) (mean ± SD; *n* = 8–9) (**a**) and glyoxalase II (GLXII) (mean ± SD; n = 4) (**b**) activities, d-LDH expression (mean ± SD; *n* = 3) (**c**), and mitochondrial d-lactate oxidation (mean ± SD; *n* = 3) (**d**) in *frostbite1* (*fro1*) as compared to wild-type (WT_*C24*). Statistically significant differences by ANOVA (*p* ≤ 0.05) with Tukey’s post hoc test are indicated by different letters above the bars

Since glycolysis is a major route of MG synthesis, MG concentration in nitrate-grown *fro1* plants was more than twice as high as that in WT plants fed with nitrate. Surprisingly, in *fro1* plants nourished with ammonium, MG concentration was only 25% of the level found under nitrate nutrition (Fig. 6d)*.* Elevated levels of MG need to be metabolized and usually induce the glyoxalase pathway activity. Indeed, GLXI activity increased by approximately 40% and 75% in nitrate-grown *fro1* plants and ammonium-grown WT plants, respectively (Fig. 7a). The highest GLXI activity was observed in *fro1* plants fed with ammonium, which was double that of nitrate-grown WT plants (Fig. 7a). Furthermore, we analyzed the transcript levels of GLXI isoforms induced in response to ammonium (Borysiuk et al. 2018). The transcript level of GLXI.3 was similar in *fro1* and WT plants during nitrate nutrition but was significantly increased in response to ammonium treatment in both genotypes (Supplementary Figure 3). Toxic SLG, formed due to GLXI activity, is subsequently a substrate for GLXII. The GLXII activity almost doubled in *fro1* compared to that in WT during nitrate feeding. Ammonium nutrition led to further induction of GLXII activity to a similar level in both genotypes (Fig. 7b). GLXII was also observed to be upregulated at the transcriptional level. In *fro1* and ammonium-grown WT plants, expression of *GLXII.4*, encoding both mitochondrial and chloroplastic GLXII isoforms was found to be induced. Additionally, in ammonium-fed *fro1*, cytosolic *GLXII.2* and mitochondrial/chloroplastic *GLXII.5* were significantly upregulated (Supplementary Figure 3). Complex I dysfunction and ammonium nutrition of WT plants did not affect *D-LDH* expression. However, *D-LDH* expression was induced in *fro1* grown under ammonium conditions (Fig. 7c). The oxygen uptake by isolated mitochondria with d-lactate as a substrate was significantly lowered in WT plants grown on ammonium, similar to our previous results (Borysiuk et al. 2018), while in *fro1* plants, it remained unchanged.

**Fig. 8:**
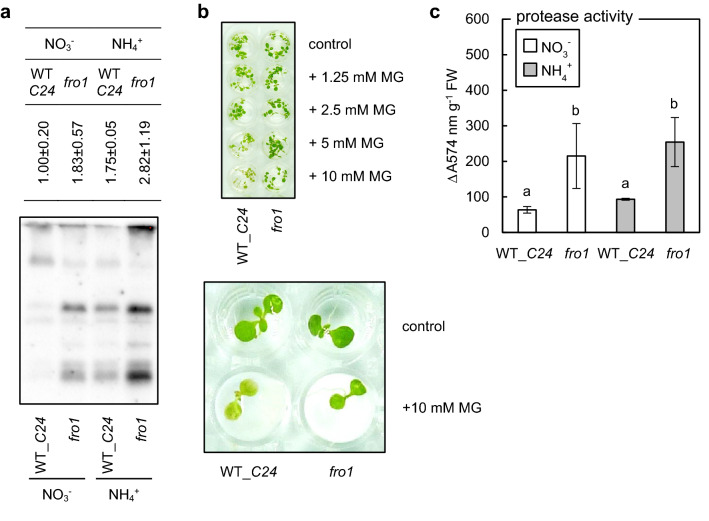
Tolerance to methylglyoxal (MG)-induced cellular damage in *Arabidopsis* plants overcoming ammonium syndrome that were grown on nitrate (NO_3_^−^) or ammonium (NH_4_^+^) as the sole nitrogen source. The level of methylglyoxal-derived hydroimidazolone 1 (MG-H1) in *frostbite 1* and wild-type (WT_*C24*) leaf tissues (**a**). Phenotypic effect of external application of methylglyoxal (MG) to *frostbite 1* and WT_*C24*) seedlings (**b**). The activity of proteolytic enzymes in *frostbite1* (*fro1*) leaf tissues as compared to WT_*C24* (mean ± SD; *n* = 3) (**c**). The signal intensity for the whole lanes quantified by densitometry (mean ± SD; *n* = 3) is given in the table above the representative protein gel blot. Statistically significant differences by ANOVA (*p* ≤ 0.05) with Tukey’s post hoc test are indicated by different letters above the bars

Next, confirming our previously published results (Borysiuk et al. 2018), we observed an increase in MG-H1 levels in WT plants under ammonium nutrition (Fig. 8a). In addition, the disruption of mitochondrial Complex I resulted in increased levels of MG-dependent protein injuries when plants were grown in control conditions on nitrate supply and even more so when *fro1* plants were grown in the presence of ammonium ions (Fig. 8a). To assess how *fro1* plants cope with exposure to MG, we tested the sensitivity of seedlings to externally applied MG. MG treatment caused chlorosis in WT seedlings, whereas *fro1* seedlings sustained normal greening even when treated with 10 mM MG (Fig. 8b). Enhanced resistance to MG treatment might be linked with efficient removal of MAGE, confirming that protease activity was highly increased in response to mitochondrial dysfunction in *fro1* plants and was not significantly modified by the nitrogen source in the media (Fig. 8c).

### Antioxidant systems capacity of *fro1* plants

**Fig. 9:**
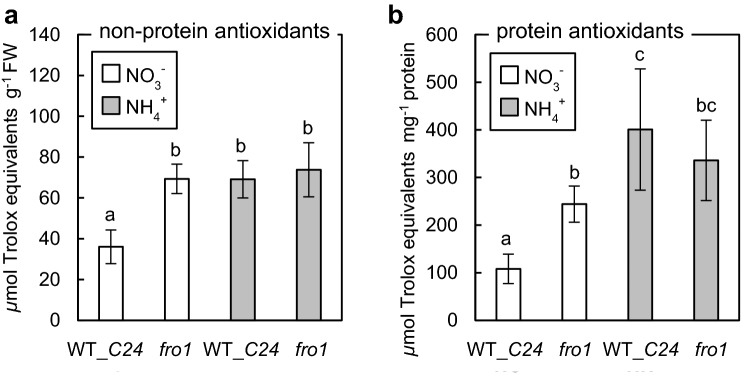
The capacity of protein (**a**) and non-protein (**b**) antioxidant systems in in *frostbite 1* and wild-type (WT_*C24*) leaf tissues. Data are presented as mean ± SD (*n* = 3). Statistically significant differences by ANOVA (*p* ≤ 0.05) with Tukey’s post hoc test are indicated by different letters above the bars

Lack of Complex I activity is a severe stress for plants that lead to cellular oxidation–reduction imbalance (Podgórska et al. 2015); therefore the antioxidative systems, both protein and non-protein based in *fro1* plants were elevated as compared to WT plants grown on nitrate (Fig. 9a, b). The additional stress factor of ammonium nutrition did not significantly modify antioxidative capacity of *fro1* leaf tissue.

## Discussion

### The glyoxalase pathway is an essential metabolic route affecting plant sensitivity to ammonium nutrition

We found that chlorosis and retarded growth rate of seedlings were principally observed in ammonium-grown plants in response to glyoxalase inhibitor treatment (Fig. 1a), or due to an increase in endogenous MG content induced by genetic disruption of the MG detoxification pathway (Fig. 1c, d). Furthermore, these phenotypic disorders matched with the enhanced MAGE concentrations in ammonium-stressed plants (Fig. 4a). Our findings fit with the observations made by other research groups, indicating that MG metabolism is an essential but still underestimated component of the stress syndrome that occurs in response to adverse environmental factors and that the efficiency of the glyoxalase pathway may be a crucial determinant of stress resistance (Kaur et al. 2014; Sankaranarayanan et al. 2017; Hasanuzzaman et al. 2017 and references therein). Increased MG levels have been found to be a consequence of salinity (Reddy and Sopory 2002;Kaur et al. 2017; Batth et al. 2020; Fu et al. 2021), drought, heavy metals, and low temperatures (Hossain et al. 2009; Gupta et al. 2018).

The differences of crop cultivars in their resistance to stress were also indicated to be correlated with accession-dependent efficiency of the glyoxalase systems and thus with tissue levels of MG (Hossain et al. 2016). It has also been shown that genetically forced increases in expression of *GLXI* and/or *GLXII* (Singla-Pareek et al. 2003, 2006; Saxena et al. 2011; Devanathan et al. 2014; Zeng et al. 2016; Batth et al. 2020) may significantly improve plant stress resistance and could help in the development of crop varieties resistant to environmental stresses (Gupta et al. 2018). In line with this, a decrease in the expression of genes encoding glyoxalases has been shown to result in a higher sensitivity to stress (Yadav et al. 2005). However, silencing/downregulation of one of several GLXI/II genes may not be sufficient to allow observation of the phenotypic effects. Our results indicated that although *GLXII.5* expression was enhanced by ammonium (Borysiuk et al. 2018), its disruption may be compensated by other GLXII isoforms and lowered GLXII activity was not observed (Fig. 2b). In contrast, disruption of *GLXI.3* cannot be fully compensated by other GLXI isoforms under ammonium stress (Fig. 2a). Also, our results indicated that the efficiency of the glyoxalase pathway is an important factor influencing plant growth under ammonium stress. The growth of *glxI.3* mutants under ammonium stress conditions was stunted (Fig. 1d). Under nitrate conditions, when MG production is low, the decreased activity of the GLXI did not lead to phenotypic changes.

It is known that the protein product of *GLXII.4*, similar to *GLXII.5*, is localized in both chloroplasts and mitochondria (Schmitz et al. 2017). Therefore, its activity could be redundant for the impaired GLXII.5. In general, cellular GLXI capacity seems to be a major determinant of resistance toward MG; GLXII and D-LDH overexpression was shown to have a lesser effect on resistance to dicarbonyl stress (Jain et al. 2018). When analyzing cellular responses to stress factors, the organellar specificity of glyoxalases should always be considered (Kaur et al. 2017; Schmitz et al. 2017). We have shown that GLXI.3 is a key player in the cellular response to ammonium stress (Fig. 2a). However, other stress factors may require the enzymatic efficiency of other differently located GLXI isoforms depending on the type of stress and the associated subcellular-localized response. In comparing the results of MG metabolism in *Arabidopsis* obtained by different research groups, special attention should be paid to the differences in the nomenclature of the particular isoforms (Mustafiz et al. 2011; Batth et al. 2020 versus Schmitz et al. 2017). In this study, we consistently followed our previous publication (Borysiuk et al. 2018) using the nomenclature of glyoxalases, as indicated by Schmitz et al. (2018). Based on the results of Batth et al. (2020), it may be presumed that GLXI.3 (named by Batth and co-workers as GLY12) activity plays the greatest role in determining the resistance to enhanced MG levels. In contrast, the decline in GLXI.2 seems to not affect stress tolerance (Tuomainen et al. 2011).

We analyzed the expression and activity of glyoxalases (Supplementary Figure 3, Fig. 2) and the level of MG-related protein injury (Fig. 4a) to better understand the development of dicarbonyl stress under ammonium nutrition. Ammonium treatment results in massive accumulation of MG-H1 protein modification, and disruption of *GLXI.3* leads to a further increase in MG-dependent damage under such conditions (Fig. 4a), which was correlated with stunted *glxI.3* plant root length (Fig. 1d). Based on these observations, it can be concluded that MAGE accumulation is a key component of ammonium stress. However, in ammonium-grown *dldh* plants, we also detected increased MAGE levels (Fig. 4a), but no growth restriction was observed (Fig. 1b). In *glxI.3* plants, a massive accumulation of MG is expected. In contrast, in *dldh* plants, d-lactate should accumulate, but MG content is probably also enhanced. The high cytotoxicity of MG is related to protein destruction (Rabbani et al. 2020a, b) and the strong mutagenic effect of MG (Thornalley 2008). d-Lactate seems to be less toxic to plants (Welchen et al. 2016) and its detrimental effect is mainly associated with cellular acidosis (Pohanka, 2020).

We did not observe a significant influence of ammonium nutrition on the expression of the D, E, and F isoforms of DJ-1 protein; however, *DJ-1A* expression was significantly upregulated (Fig. 3a). Considering previous studies indicating a physiological role for DJ-1C (Lin et al. 2011) and its lack of affinity toward MG (Kwon et al. 2013), we did not analyze the role of this protein. Additionally, since *DJ-1E* and *DJ-1F* expression was not modified by ammonium (Fig. 3a), and their glyoxalase activity was very low (Kwon et al. 2013) due to the evolutionary loss of catalytic C-end residues (Ghosh et al. 2016), we did not consider insertional mutants with disruption of these genes. Although *DJ-1A* and *DJ-1B* expression was upregulated by ammonium (Fig. 3a), the disruption of both genes did not significantly affect the root length of ammonium-treated *Arabidopsis* plants (Fig. 3c). It was previously shown that *Arabidopsis DJ-1B* mainly plays the role of holdase (Xu et al. 2010). Its glyoxalase activity is inhibited by ROS (Lewandowska et al. 2019). As long-term ammonium treatment results in increased ROS levels (Podgórska et al. 2013), the glyoxalase activity of DJ-1B may be lost under such conditions. Animal DJ-1 proteins protect against oxidative stress by acting as transcriptional regulators of antioxidative gene batteries (Kahle et al. 2009) or by stabilizing antioxidant enzyme activity (Chin et al. 2021). DJ-1 may also serve a similar function in plants because it has been shown that *Arabidopsis* insertional mutants possessing elevated DJ-1A levels are characterized by increased protection against environmental stress conditions achieved through DJ-1A-dependent activation of ROS-quenching enzymes, including superoxide dismutase 1 (SOD1) and glutathione peroxidase 2 (GPX2) (Xu et al. 2010), or interaction with ascorbate peroxidase 1 (APX1) (Xu and Møller 2010). *Arabidopsis* DJ-1A is localized to the cytosol and the nucleus (Xu et al. 2010) and its upregulated expression may be related to an increased demand for enhanced antioxidant capacity under ammonium feeding conditions when oxidative stress occurs (Podgórska et al. 2018a, b). Although DJ-1D displays the most distinct glyoxalase activity among all the DJ-1 proteins (Kwon et al. 2013), neither upregulation of DJ-1D by ammonium (Fig. 3a) nor modification of the root length of the *dj-1d* plants was observed (Fig. 3c). Consequently, we ruled out the involvement of DJ-1 proteins in defense against the harmful effects of MG in ammonium-grown *Arabidopsis*.

We have proven that MG-dependent damage to biomolecules contributes to ammonium syndrome symptoms. Our results suggested that the classical MG detoxification pathway, based on GSH-dependent glyoxalases, plays a crucial role in the plant response to ammonium nutrition.

### Differences in ammonium resistance of *fro1* plants may be a consequence of the enhanced GLXI activity and mtETC capacity to accept electrons

Since *fro1* plants do not show typical growth retardation under ammonium nutrition (Fig. 6a), we analyzed the modification of methylglyoxal metabolism in this genotype. In *fro1* plants grown on ammonium, glycolytic flux is more balanced than in WT because NADP^+^-GAPDH activity is elevated (Fig. 6c), which can counteract the accumulation of TP, thereby lowering MG production under ammonium treatment. Another adaptation of *fro1* to control MG release from TP appears to be the induction of TPI in *fro1* plants (Fig. 6b). Admittedly, TPI activity is considered a source of MG formation. However, it has been suggested that because the TPI structure stabilizes the binding of the intermediate of the interconversion of DHAP and GAP, its engagement in the reaction significantly reduces MG formation (Dorion et al. 2021).

Additionally, GLX activity was most pronounced in these plants (Fig. 7a, b), and the following changes resulted in lowered MG levels (Fig. 6d). However, the greatest differences between ammonium-grown WT and *fro1* plants were displayed in the final step of MG detoxification, in contrast to WT plants. In the latter case, the ability of d-lactate oxidation by mtETC was significantly decreased (Borysiuk et al. 2018). However, *fro1* plants maintained a stable d-lactate oxidation rate (Fig. 7d) and were more resistant to exogenous d-lactate (K. Borysiuk, B. Szal, results not presented). Such a feature seems not to be a simple consequence of the differences in the level of electron acceptor for reaction by d-LDH since cytochrome *c* (cyt *c*) levels are lower in ammonium-grown *fro1* plants than in WT plants (Podgórska et al. 2018a, b). However, d-LDH function requires oxidized cyt *c* availability, and its level depends on the electron loading process and forwarding toward Complex IV of the mtETC. Increased cyt *c* oxidase (COX) capacity and a significant increase in alternative oxidase (AOX) protein levels is characteristic of *fro1* plants (Podgórska et al. 2015), which may lead to an increase in oxidized cyt *c* availability, thereby allowing more efficient d-lactate oxidation. Additionally, it is tempting to assume that the lack of ammonium growth inhibition is related to the connection between respiratory chain function and DJ-1 proteins. Previous studies suggest that DJ-1 can translocate to mitochondria in human cells after cysteine 106 residue ROS-dependent oxidation (Canet-Avilés et al. 2004). The sulfinated DJ-1 interacts with Complex I to maintain its activity (Hayashi et al. 2009). In addition, DJ-1 lowers mitochondrial ROS synthesis, triggering the expression of nuclear genes encoding uncoupling proteins (UCP) (Dolgacheva et al. 2019 and references therein). The translocation of DJ-1 homolog, yeast HSP31 protein, into plant mitochondria in response to stress conditions has also been reported (Melvin et al. 2017). It is likely that DJ-1 could also reverse MG-derived glycation of proteins and DNA as demonstrated in mammalian cells (Richarme et al. 2015, 2017; Zheng et al. 2019). However, it is still controversial whether its deglycase activity was not mistaken (Andreeva et al. 2019) and even if DJ-1 had such a dual function in combating MG deleterious effects, it would be of physiological relevance in plants. Our results obtained thus far do not indicate a significant role of DJ-1 proteins under ammonium stress conditions (Fig. 3). However, such a role cannot be excluded because we have not tested the role and localization of DJ-1 proteins in *fro1* plants, and its role may be modified in plants with dysfunctional mitochondria.

We also showed that *fro1* plants are more resistant to MG and tolerate higher MAGE levels (Fig. 8a) without visible phenotypic disorders (Fig. 8b). Studies indicate that several hundred plant proteins undergo glycation under both non-stress (Bilova et al. 2016, 2017) and stress conditions (Paudel et al. 2016). It was proposed that similar to animal systems, plant glycation of particular proteins (Bilova et al. 2017) may mark those biomolecules for degradation (Shumilina et al. 2019), enabling the likely redistribution of nutrients. Although glycation represents one of several non-enzymatic modifications marking proteins toward proteolysis, it seems to be characterized by high specificity since the overlap between the ROS-oxidized and glycated proteins is very low (Matamoros et al. 2018). The proteolytic activity of *fro1* plants was highly enhanced (Fig. 8c), which may contribute to the very efficient removal of glycated proteins under ammonium feeding conditions, despite the higher MG-H1 level (Fig. 8a). Increased protease activity in *fro1* tissue is possibly also engaged in the removal of ROS-injured proteins. The link between increased protease activity and dysfunction of respiratory metabolism in *fro1* plants needs further studies. Despite the fact, that mitochondrial Lon1 is believed to selectively degrade oxidatively damaged proteins (Bota and Davies 2002) the elevated protein oxidation was not found in *lon1* mutants (Solheim et al. 2012). Besides, *fro1* plants seem to have a very efficient system of protection against ROS (Fig. 9). Reductive stress caused by mitochondrial dysfunction is a sufficient factor for anti-ROS defense system activation in *fro1* plants grown under control conditions. Previously an activation of the antioxidant system because of Complex I dysfunction was also shown in other plant species that was associated with enhanced stress tolerance (Juszczuk et al. 2012 and references therein). In *fro1* plants due to an efficient antioxidative system and enhanced system removal of ROS-injured biomolecules neither an increase in H_2_O_2_ nor enhanced protein carbonylation (Podgórska et al. 2015) was observed.

Using *fro1* as a specific model plant characterized by the enhanced growth rate in response to ammonium (Podgórska et al. 2015), we have shown that besides the enhanced activity of glyoxalases, the efficient mitochondrial step of d-lactate oxidation may determine resistance to ammonium treatment. This finding is consistent with previous reports, indicating that GLXI and d-LDH are critical players in methylglyoxal detoxification under oxidative stress (Jain et al. 2018).

### MG may modify the anti-ROS defense in *Arabidopsis* leaf tissue, but it does not result in significant amplification of oxidative stress under long-term ammonium nutrition

Previously, we reported that ammonium treatment of WT plants resulted in increased ROS production (Podgórska et al. 2013, 2015) and an increase in antioxidant defense capacity (Fig. 5a, c, d). In this study, we have shown that the disruption of the MG detoxification pathway under ammonium conditions restricts anti-ROS defense because a considerable decrease in the capacity of protein antioxidants was observed (Fig. 5d). Although the analysis of non-protein defense systems has not revealed such a drop (Fig. 5c), its efficiency relies on enzyme-dependent regeneration (Foyer and Noctor 2011) and can be impaired under prolonged stress. Our results indicated that ROS and MG metabolism were closely related in non-stress conditions because the disruption of GLXI led to enhanced capacity of both non-enzymatic and enzymatic antioxidant systems under control growth conditions (Fig. 5c, d). The boosted ROS defense in nitrate-grown *glxI.3* plants points out that MG, when present at a low concentration, plays the role of a messenger molecule in plant tissue, as postulated by Hoque et al. (2016).

The signaling role of MG is well documented in yeast systems under osmotic stress, where it triggers the high-osmolarity glycerol (HOG)-mitogen-activated protein (MAP) kinase cascade (Maeta et al. 2005). In animal systems, the receptor for AGE (RAGE) was discovered in the 1990s (Schmidt et al. 1992) and the signaling pathway initiated by the binding of AGEs to the receptor has been studied in detail (Xue et al. 2014). The plant research on the signaling role of MG or MG-related metabolites is still in its preliminary stages. However, it was shown that MG treatment of plants significantly alters the rice transcriptome: the expression of signaling protein kinases and proteins involved in stress adaptation was greatly upregulated, and the specific MG-responsive element was identified (Kaur et al. 2015). It remains unclear whether, at a fourfold increase due to ammonium supply (Borysiuk et al. 2018) in WT plants, MG acts mainly as a signaling molecule or a certain quantitative threshold is exceeded and MG-dependent damage to biomolecules restricts the antioxidant system functioning. Both situations are possible in WT ammonium-treated plants, because of the increased MG-H1 modifications (Fig. 4a) together with the enhanced capacity of the enzymatic antioxidant and proteolytic system (Fig. 5d, Borysiuk et al. 2018, Supplementary Figure 2) and increased levels of ROS-dependent protein damage (Fig. 5b). However, considering that its concentration in *Arabidopsis* WT plants grown under ammonium nutrition was around 4 µM (Borysiuk et al. 2018) and that the inhibitory effect of MG toward antioxidant enzymes was observed at much higher concentrations (at least 500 µM; Hoque et al. 2010, 2012), it seems that the enzymatic antioxidant system should not be significantly impaired in ammonium-grown WT plants. Previously, it was shown that the concentration of MG in stressed plant tissues may be in the range of 100–300 µM without inhibitory effects toward enzyme activity (Yadav et al. 2005). It is likely that in *Arabidopsis* plants with impaired glyoxalase pathway under ammonium stress, MG content reached a high level, impairing the antioxidative activity of enzymes (Fig. 5d). Additionally, it may also be presumed that in *vivo*, the decreased activity of GLXI in *glxI.3* plants, together with increased MG production, may result in spontaneous trapping of reduced glutathione in hemithioacetal molecules (Deponte 2013).

The degree of proteome glycation in different cellular compartments was distinct. In *Arabidopsis* grown under control conditions, the level of MG-H1 in mitochondrial proteins was lower compared to that in chloroplasts (M. Ostaszewska-Bugajska, unpublished results). Interesting results concerning the impact of stress-induced glycation on plant metabolism were recently presented by Chaplin et al. (2019). Among the core 112 proteins targeted for glycation in *Arabidopsis* leaves in response to abiotic stress, a large group was identified as oxidoreductases. Besides chloroplast-localized proteins, which are most probably associated with sugar and MG metabolism in these organelles, glycation mainly involves apoplast-localized proteins (Chaplin et al. 2019). Since no sugar or MG is produced in the extracellular space, the reason for the enhanced glycation of apoplastic proteins in response to stress might be a specific regulatory process resulting in the modification of the activity of enzymes localized in this compartment, such as the enzymes engaged in cell wall rearrangement. In turn, this can lead to stress-responsive growth restrictions. Previously, we showed that modified apoplast metabolism, with particular regard to extracellular ROS and cell wall metabolism (Podgórska et al. 2017; Burian et al. 2022), is a crucial factor determining ammonium-induced growth retardation. However, the level of glycation of apoplastic proteins under ammonium nutrition has not yet been evaluated.

**Fig. 10:**
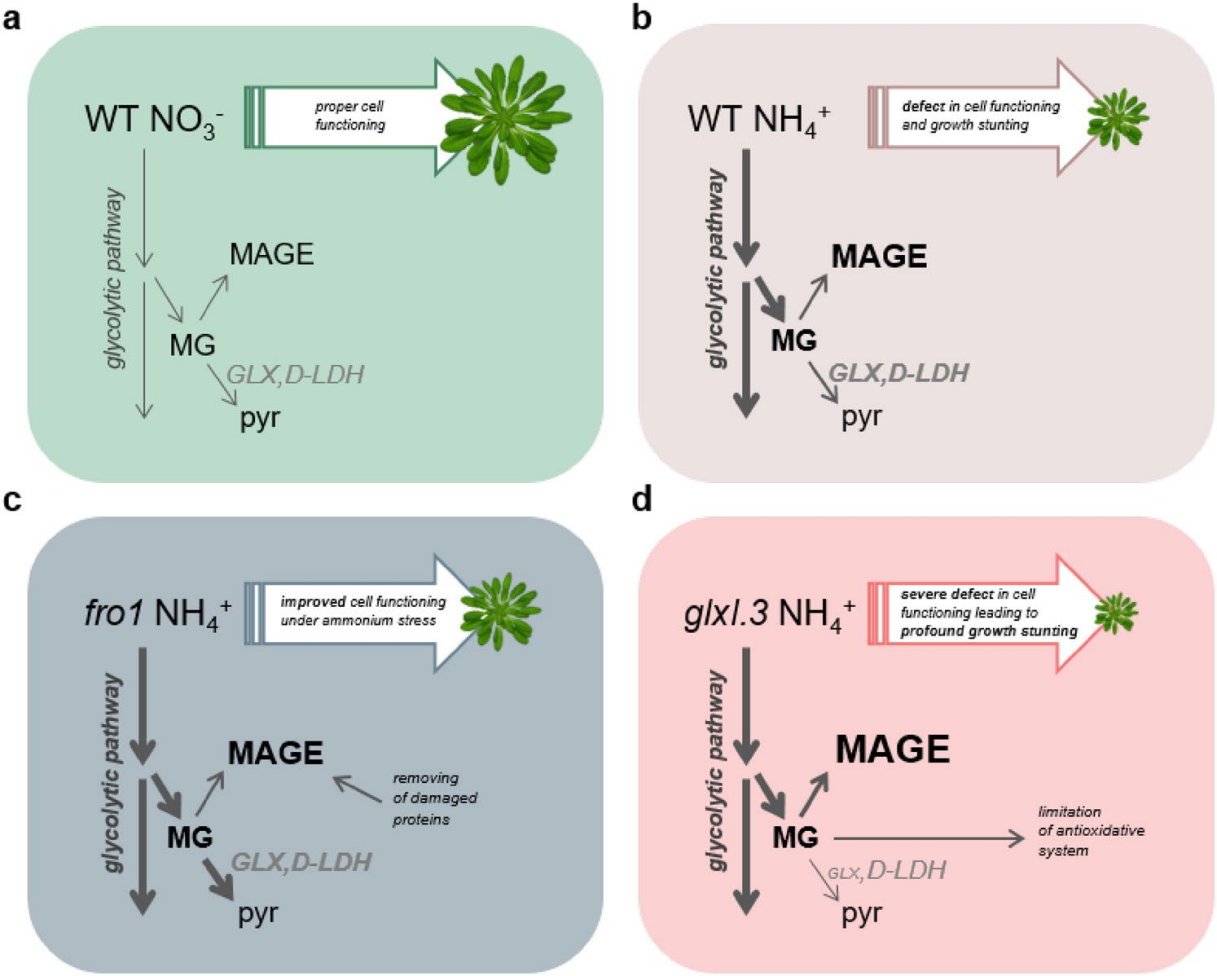
Scheme showing the role of methylglyoxal (MG) metabolism under conditions of ammonium stress. MG metabolism in nitrate-grown wild-type (WT) plants (**a**), ammonium-grown wild-type plants (**b**), and mutants *frostbite 1* and *glx1.3* grown under conditions of ammonium stress, respectively (**c**) and (**d**)

In conclusion, the canonical pathway for MG detoxification, based on GSH-dependent glyoxalases, is crucial under ammonium nutrition. A key enzyme in this route is GLXI, and impairment of its activity leads to enhanced *Arabidopsis* rosette growth inhibition and leaf chlorosis. Our results indicated that glyoxalase III (DJ-1) is of marginal importance under ammonium nutrition. Furthermore, we demonstrated that the accumulation of MAGE is an essential component of ammonium stress (Fig. 10). Using *fro1* as a specific model plant, we showed that the enhanced growth rate in response to ammonium nutrition is accompanied by a more resistant phenotype to MG and tolerance to elevated MAGE levels. However, the mechanism linking the lack of active Complex I with MG tolerance remains obscure. We have also shown that the efficiency of glyoxalase activity is an important factor that influences the operation of cellular antioxidative systems. Disruption of the MG detoxification pathway under ammonium stress leads to restriction of the anti-ROS defense, mainly by inhibition of protein antioxidants. Furthermore, our results confirmed that MG functions as a messenger molecule that induces antioxidant systems in plants grown under non-stress conditions.

## Supplementary Information

Below is the link to the electronic supplementary material.

Supplementary file1 (PDF 578 KB)

## Data Availability

All data supporting the findings of this study are available within the article and the associated Supplementary Information files.
