# Supplementary material for: Glyoxalase I activity affects Arabidopsis sensitivity to ammonium nutrition
Source: Plant Cell Rep. 2022 Oct 15;41(12):2393–413. doi: 10.1007/s00299-022-02931-5 (PMC9700585; doi:10.1007/s00299-022-02931-5)
Supplement: Supplementary file 1 — Supplementary file1 (PDF 578 KB) [file 299_2022_2931_MOESM1_ESM.pdf]

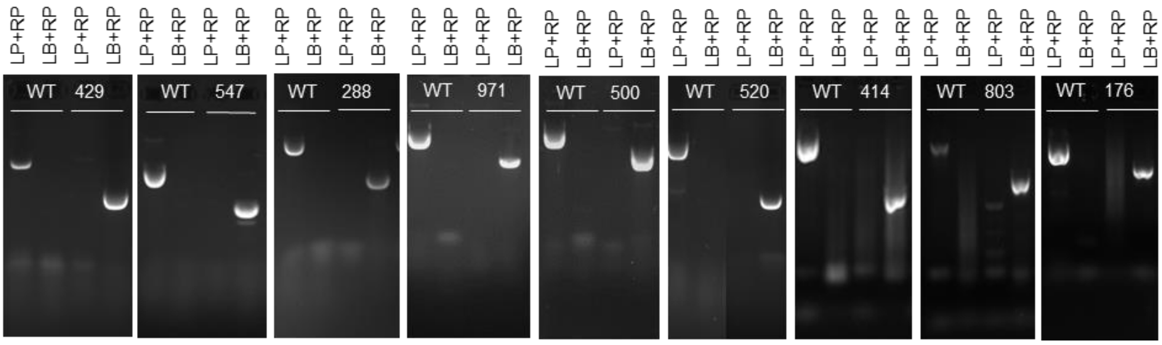


**Supplementary Fig. 1** Image of gels after separation of products amplified by PCR reaction using specific primers during genotyping of insertional mutants. WT (WT_*Col-0*); 429, *glxI.3_429* (N535429 line); 547, *glxI.3_547* (N631547 line); 288, *glxII.5_288* (N514288 line); 971, *glxII.5_971* (N662971 line); 500, *d-ldh_500* (N554500 line); 520, *d-ldh_520* (N685520 line); 414, *dj-1b* (N593414); 803, *dj-1a* (N500803); 176, *dj-1d* (N659138). Here, LP: left primer; RP, right primer; LB, left border primer of the T-DNA insertion (seq.: GCGTGGACCGCTTGCTGCAACT). LB primer is the same for all genotyped insertional mutants.

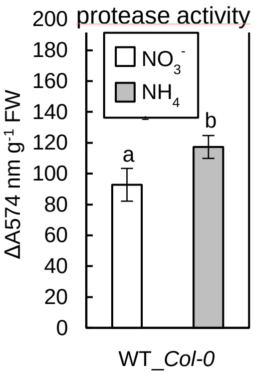


**Supplementary Fig. 2** Activity of proteolytic enzymes assayed with the universal protease substrate in wild-type (WT_*Col-0*) plants grown long-term in the presence of 5 mM nitrate ($NO_3^-$) or ammonium ($NH_4^+$). Data are means±SD (n=5). Statistically significant differences by ANOVA (p≤ 0.05) with Tukey's post-hoc test are indicated by different letters above the bars.

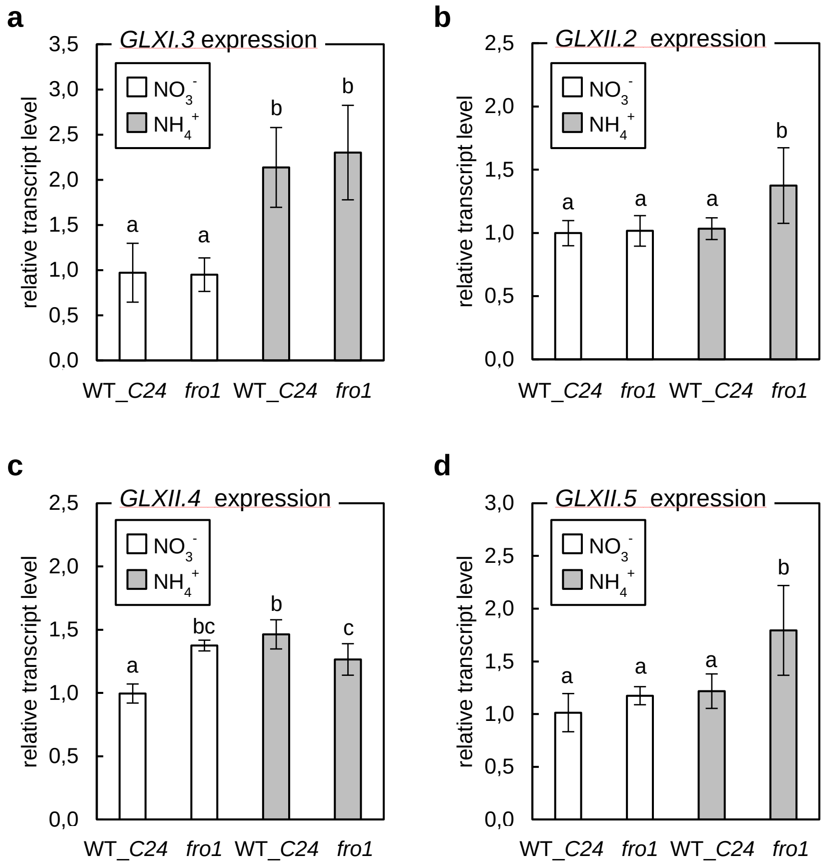


**Supplementary Fig. 3** Relative transcript level of glyoxalase (GLX) genes in *Arabidopsis thaliana frostbite1* (*fro1*) as compared to wild-type (WT_*C24*) long-term grown plants in the presence of 5 mM nitrate ($NO_3^-$) or ammonium ($NH_4^+$). The relative fold changes were calculated to the value for wild-type (WT) plants grown on $NO_3^-$, which is presented as 1. Data are means±SD (n=5). Statistically significant differences by ANOVA ($p \leq 0.05$) with Tukey's post-hoc test are indicated by different letters above the bars.

**Supplementary Table 1.** Primer sequences used for PCR-based genotyping of insertional mutants.

| Line | NASC ID | Left Primer (LP) | Right Primer (RP) |
|---|---|---|---|
| ***glxI.3_429*** | N535429 | 5'– GCAAGCGACTATCATCACTCC –3' | 5'– GTTCTCCATGAGCTTCGTGAG –3' |
| ***glxI.3_547*** | N631547 | 5'– TGCCCTGTGACAACAACATAG –3' | 5'– ATTCGGAAAAACCGTCTTTTG –3' |
| ***glxII.5_288*** | N514288 | 5'– TTGAGAAAGCTTCTTGTTGCC –3' | 5'– ACGTTTAGATGTGCATTTGGC –3' |
| ***glxII.5_971*** | N662971 | 5'– CAAATTCCCACCAGTGGTGATC –3' | 5'– TGGTTTAGTATTCGGCACCAC –3' |
| ***dj-1a*** | N500803 | 5'– GGTCGTTCAGAGAAGACGATG –3' | 5'– CGACAAACTTTCGGACAAGAG –3' |
| ***dj-1b*** | N593414 | 5'– AGGCACAAATTGCTCCATATG –3' | 5'– ACCATGGAATTCTCTGTCACG –3' |
| ***dj-1d*** | N659138 | 5'– GAGGGAGAGAGAAACCTGGTG –3' | 5'– TATCTTTTGTGGTTGCTTCCC –3' |

**Supplementary Table 2.** Primer sequences used for RT-qPCR assays.

| Gene | AGI identification | Forward Primer | Reverse Primer |
|---|---|---|---|
| ***DJ-1A*** | AT3G14990 | 5’– TCCAGTATCTTGCTGCTTCGTC –3’ | 5’– ACCGCCAGGTAACACAATCA –3’ |
| ***DJ-1B*** | AT1G53280 | 5’– ATTGTGTTGCCTGGTGGTCTC –3’ | 5’– TGATTCCGCCTGCTTCTTTA –3’ |
| ***DJ-1D*** | AT3G02720 | 5’– TGGAAGACTACGAGGTGATGGTT –3’ | 5’– CAGACGGTGTGGACGGTGAT –3’ |
| ***DJ-1E*** | AT2G38860 | 5’– TTGGGGACTATGTAGAAGATTATGG –3’ | 5’– CCCTCCTGGAACCACTACACA –3’ |
| ***DJ-1F*** | AT3G54600 | 5’– TGATTGGGGATTGTGTTGAAGA –3’ | 5’– GGCGTCACTGCATCCACTTTA –3’ |
| ***D-LDH*** | AT5G06580 | 5’– GAGTGCTGCTGGATACGATTT –3’ | 5’– GCAAACTGCCACCACTGAATG –3’ |
